# p38β, A Novel Regulatory Target of Pokemon in Hepatic Cells

**DOI:** 10.3390/ijms140713511

**Published:** 2013-06-27

**Authors:** Zhe Chen, Feng Liu, Nannan Zhang, Deliang Cao, Min Liu, Ying Tan, Yuyang Jiang

**Affiliations:** 1 Department of Chemistry, Tsinghua University, Beijing 100084, China; E-Mail: czseasky@163.com; 2 The Ministry-Province Jointly Constructed Base for State Key Lab—Shenzhen Key Laboratory of Chemical Biology, Graduate School at Shenzhen, Tsinghua University, Shenzhen 518055, China; E-Mails: xiaoee2728@126.com (N.Z.); liumin880608@163.com (M.L.); tan.ying@sz.tsinghua.edu.cn (Y.T.); 3 Department of Medical Microbiology, Immunology & Cell Biology, Simmons Cooper Cancer Institute, Southern Illinois University School of Medicine, Springfield, IL 62702, USA; E-Mail: dcao@siumed.edu; 4 Department of Pharmacology and Pharmaceutical Sciences, School of Medicine, Tsinghua University, Beijing 100084, China

**Keywords:** Pokemon, p38β, transcription factor, hepatic cell, SB202190

## Abstract

Pokemon is an important proto-oncogene involved in various biological processes and cancer development, such as cell differentiation, tumorigenesis and metastasis. Pokemon is recognized as a transcription factor localized upstream of several oncogenes, regulating their expression. p38MAPKs act as key regulatory factors in cellular signaling pathways associated with inflammatory responses, cell proliferation, differentiation and survival. p38β, a member of p38MAPK family, is closely correlated with tumorigenesis, but the mechanism of activation remains unclear. In this study, we found overexpression of Pokemon promoted the growth, migration and invasion of HepG2 cells. However, a p38 inhibitor SB202190 efficiently attenuated the promoting effect of Pokemon in the HepG2 cells. Targeted expression or silencing of Pokemon changed cellular p38β protein level and phosphorylation of downstream ATF2 in the p38 signaling pathway. Both dual luciferase report assay and ChIP assay suggested that p38β is a novel regulatory target of the transcription factor Pokemon and positively regulated by Pokemon in hepatic cells.

## 1. Introduction

Pokemon (POK erythroid myeloid ontogenic factor), also named as Zbtb7A, LRF, FBI-1 and OCZF, contains an *N*-terminal POZ/BTB domain mediating protein-protein interaction and a *C*-terminal Krüppel zinc finger domain responsible for DNA binding [1]. Pokemon is an important transcriptional factor with multiple biological functions, which was originally identified by specifically binding to the inducer of short transcripts (IST) element of human wild type HIV-1 [1]. Pokemon is essential for erythroid differentiation and maturation [2] and fate decision of B versus T lymphoid lineage [3]. Pokemon interacts with POZ family members to form a homo- or hetero-dimer participating in various biological processes through its POZ/BTB domain [4]. Pokemon-deficient mouse embryonic fibroblasts (MEFs) are completely unresponsive to either proliferative stimuli or transformative signals of oncogene combinations [5,6].

Pokemon is overexpressed in multiple carcinomas [7–10] and triggers abnormal expression of effector genes and related signaling [11]. Pokemon acts as a transactive or suppressive transcription factor [12,13], inhibiting p53 (mediated through the ARF-Mdm2-p53 pathway) [5] and Rb gene transcription [14] but promoting NF-κB transcription and nuclear localization [15,16]. Therefore, Pokemon plays a pivotal role in tumorigenesis as a proto-oncogene localized upstream of several oncogenes [17].

p38MAPKs (p38 mitogen-activated protein kinases) are MAPK family members and key regulatory factors in cellular signaling pathways involved in inflammatory response and cell proliferation, differentiation, survival, migration and invasion [18–21]. Consistent with the importance of these events in tumorigenesis, p38MAPK signaling is associated with human cancers. p38MAPKs are phosphorylation-activated by environmental stresses and mitogens, such as UV irradiation, inflammatory cytokines and growth factors [22], followed by activation of a series of signaling proteins in the pathways and leading to relative biological events. Among the downstream proteins, MSK1, MAPKAPK2 and ATF2 are direct substrates of p38 [23–25].

p38MAPKs consist of 4 members: p38α, p38β, p38γ and p38δ, which are encoded by four different genes. p38α and p38β are ubiquitously expressed in most cell types, while p38γ and p38δ have more restricted expression patterns and specialized functions [23]. p38β encoded by the MAPK11 gene shows the highest sequence identity to p38α, but they share distinct substrate affinity and biological functions [26]; their signaling regulation is different [27,28]. p38β emerges at low levels in most cells and tissues, and its contribution to tumorigenesis is not fully clear [29,30].

In this study, we found that a p38 inhibitor SB202190 efficiently attenuated the promoting effect of Pokemon on cell growth, migration and invasion in the HepG2 cells. Consequently, we specifically targeted Pokemon expression in different hepatic cell lines and estimated the effect on the expressions of p38α, p38β and phosphorylated downstream signaling proteins ATF2. Finally, we identified p38β as a new regulatory target of Pokemon in hepatic cells, which is perhaps a new mechanism through which Pokemon is involved in different biological processes.

## 2. Results and Discussion

### 2.1. Pokemon Promotes HepG2 Cell Growth, Migration and Invasion

**Figure 1:**
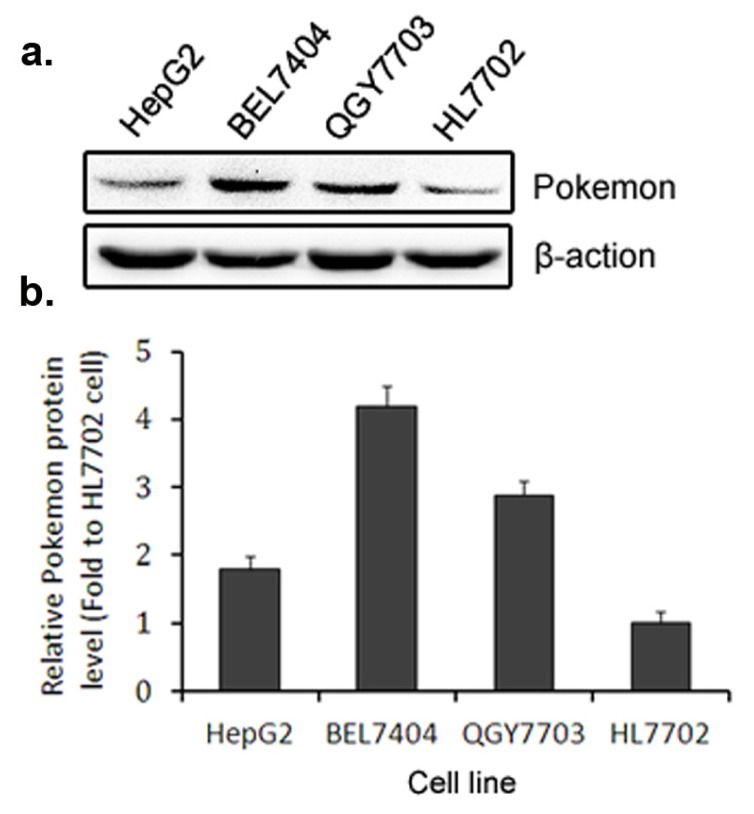
Endogenous Pokemon expression in different hepatic cell lines. (**a**) Western blot in which cells were lysed and total proteins were collected in HepG2, BEL7404, QGY7703 and HL7702 cells, respectively; (**b**) Quantification of western blot data.

We examined Pokemon expression in several hepatic cell lines and found the expression level of Pokemon is correlated with the malignancy of hepatic cells (Figure 1). The protein level of Pokemon is high in malignant hepatocarcinoma cells BEL7404, but low in immortalized hepatic cells HL7702. HepG2 cells were derived from a hepatoblastoma, but are not tumorigenic in nude mice, in which Pokemon is expressed at a moderate level.

**Figure 2:**
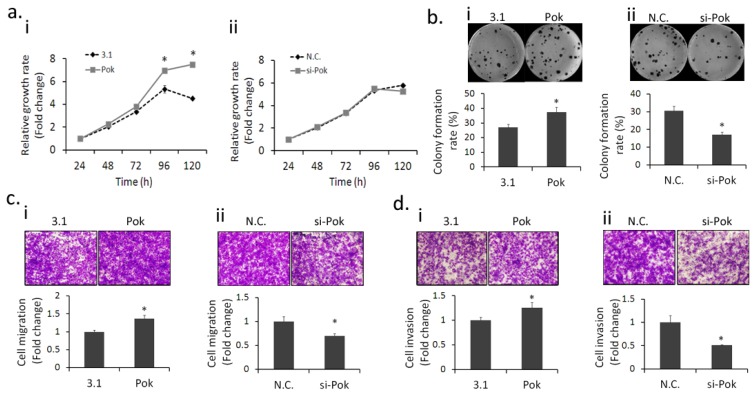
Pokemon promotes HepG2 cell growth and metastasis. (**a**) HepG2 cell growth rate: i. Cells were transfected with pcDNA3.1(−)-Pokemon (marked as Pok) or pcDNA3.1(−) (marked as 3.1); ii. Pokemon was silenced by si-RNA (marked as si-Pok) or scramble RNA (marked as N.C.) in HepG2 cells; (**b**) Effect of Pokemon on colony formation in HepG2 cells: i. Cells were transfected with pcDNA3.1(−)-Pokemon or pcDNA3.1(−); ii. Pokemon was silenced by si-RNA or scramble RNA in HepG2 cells, Bar chart below the photo stands for the colony formation rate (%). The colony formation rate stands for the proportion of final clone number accounted for in plated cell number; (**c**) *In vitro* migration assays: Pokemon was overexpressed (i) or silenced (ii) in HepG2 cells; (**d**) *In vitro* invasion assays. Pokemon was overexpressed (i) or silenced (ii) in HepG2 cells. Bar chart below the photo stands for the relative fold of the migrated or invaded cell number compared to the negative control group. * *p* < 0.05 compared to the negative control group.

It is reported that Pokemon acts as an oncogene and plays a critical role in tumorigenesis [5]. Hence, we wondered what were the biological effects of Pokemon in hepatic cells. For better comparison, we chose HepG2 cells with a moderate endogenous Pokemon level for ectopic expression or silencing manipulation, and cell growth was estimated by MTT assays. Our results showed that ectopic expression of Pokemon significantly promoted HepG2 proliferation (Figure 2a(i)); however, Pokemon silencing did not notably affect HepG2 cell proliferation (Figure 2a(ii)). To further confirm this finding, we up-regulated Pokemon expression in immortalized hepatic cells HL7702 and silenced Pokemon in hepatocarcinoma cells BEL7404, and obtained consistent results (Figure S1).

We further examined the effect of Pokemon on clonogenic growth in HepG2 cells and the results showed Pokemon overexpression notably enhanced the number of colony formations (Figure 2b(i)), and inversely, the silencing of Pokemon significantly inhibited colony formation (Figure 2b(ii)). Because of population dependence, the growth behavior of single cells in colony formation is extremely different from the proliferation of massive cells in the MTT assay.

A main characteristic of malignant cells is the invasive and metastatic ability [31]. To understand the role of Pokemon in HepG2 cell metastasis, we conducted transwell migration and invasion assays. The results demonstrated that Pokemon overexpression significantly promoted the migration (Figure 2c(i)) and invasion (Figure 2d(i)) of HepG2 cells while silencing by si-RNA reduced the migration (Figure 2c(ii)) and invasion (Figure 2d(ii)), and consistent results were obtained with using BEL7404 cells with silenced Pokemon (Figure S2).

### 2.2. p38 Inhibitor SB202190 Attenuates the Promotion of Pokemon on HepG2 Growth, Migration and Invasion

**Figure 3:**
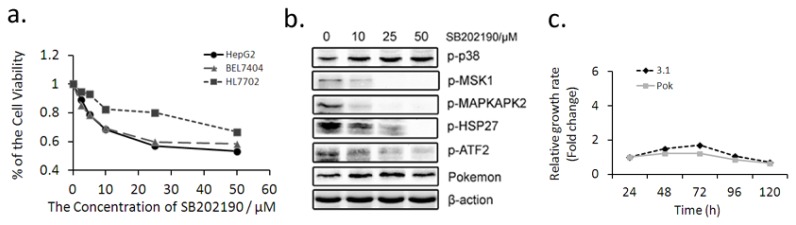

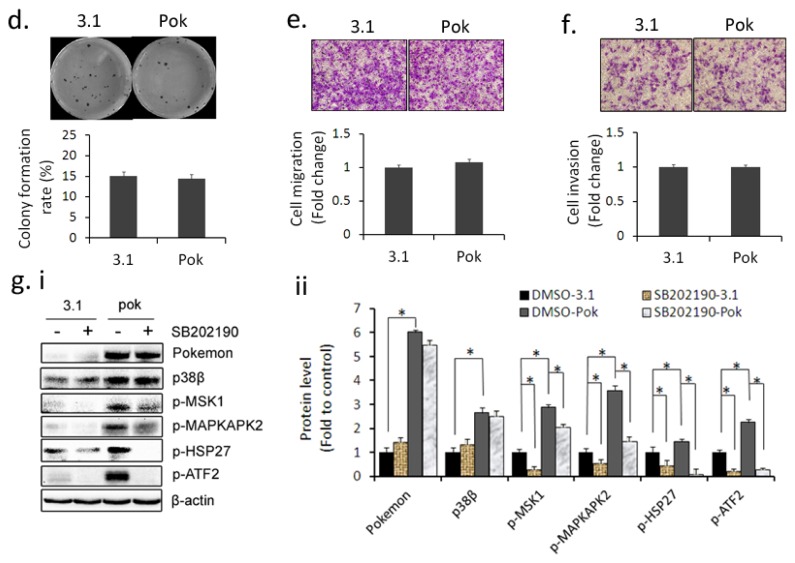
(**a**) Inhibition of SB202190 on cell viability: MTT assays in HepG2, BEL7404 and HL7702 cells treated with SB202190 for 48 h at different concentrations (0, 2.5, 5, 10, 25 and 50 μM); (**b**) Western blot: Displaying that SB202190 dose-dependently inhibits the phosphorylation of p38 downstream proteins. HepG2 cells were treated with SB202190 for 24 h at different concentrations (0, 10, 25 and 50 μM); (**c**–**g**) HepG2 cells were treated with 25 μM SB202190 at 24 h after transfecting with pcDNA3.1(−)-Pokemon or pcDNA3.1(−): (**c**) HepG2 Cell growth rate; (**d**) Effect of Pokemon and p38 inhibitor SB202190 on colony formation in HepG2 cells, the colony formation rate stands for the proportion of final clone number accounted for in plated cell number; (**e**) *In vitro* migration assays; (**f**) *In vitro* invasion assays. Bar chart below the photo stands for the relative fold of the migrated or invaded cell number compared to the negative control group; (**g**) Pokemon activates p38 signaling pathway in hepatic cells: Left panel is Western blot bands. Western blot in HepG2 cells after Pokemon was overexpressed for 60 h, and the cells were treated by SB202190 at the concentration of 25 μM; right panel is quantification of western blot data. * *p* < 0.05 compared to the negative control group.

p38 inhibitor SB202190 specifically inhibits the activity of p38β and p38α [32]. We found SB202190 had dose-dependent inhibition on the proliferation of HL7702, HepG2 and BEL7404 cells, and the inhibition on hepatocarcinoma cells BEL7404 and hepatoblastoma-derived cells HepG2 was more significant (Figure 3a). SB202190 inhibited the phosphorylation of p38 downstream proteins MAPKAPK2, ATF2, MSK1 and HSP27 in a dose-dependent manner, and the inhibition was notable at the concentration of 25 μM. However, SB202190 slightly increased the phosphorylation of p38, a result similar to that reported using its analogue, SB203580 [24], and it had no significant effect on Pokemon expression (Figure 3b).

We further found that SB202190 (25 μM) suppressed the stimulation of ectopic Pokemon on HepG2 proliferation (Figure 3c) and colony formation (Figure 3d). Similarly, SB202190 (25 μM) efficiently attenuated the stimulative role of Pokemon in cell migration (Figure 3e) and invasion (Figure 3f). Moreover, we further found Pokemon overexpression increased the phosphorylation of p38 downstream proteins in HepG2 cells, and 25 μM SB202190 effectively inhibited the phosphorylation levels with no effect on Pokemon expression (Figure 3g), which indicated that the effect of Pokemon on HepG2 cell growth and metastasis may be associated with p38 pathway.

Together these data suggested that the promotion on HepG2 growth, migration and invasion by ectopic expression of Pokemon was efficiently attenuated by 25 μM SB202190. Combined with the effect of Pokemon on p38β and the transcription factor role of Pokemon by ChIP-on-Chip assay [33], we proposed the hypothesis that the promotion of Pokemon on HepG2 cells was associated with its regulation on p38β expression.

### 2.3. Pokemon Up-Regulates p38β Expression in Hepatic Cells

**Figure 4:**
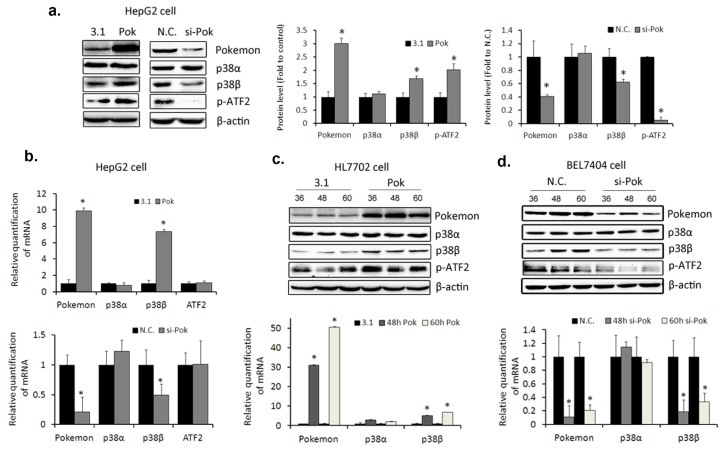
Pokemon up-regulates p38β expression in hepatic cells. Pokemon was delivered by expression plasmid pcDNA3.1(−)-Pokemon with pcDNA3.1(−) as a negative control. Pokemon silencing was triggered by si-RNA. (**a**) Targeted expression or silencing of Pokemon in HepG2 cells. Cells were collected at 60 h after transfection or silencing (Left panel). And the quantification of western blot data was displayed on the Right panel; (**b**) Real-time quantitative polymerase chain reaction (qPCR) at 48 h after transfection in HepG2 cells. Upper panel: Ectopic expression of Pokemon; lower panel: Silencing of Pokemon; (**c**) Ectopic expression of Pokemon in HL7702 cells. Upper panel: Western blot in which cells were lysed and total proteins were collected at 36, 48, 60 and 72 h, respectively. Lower panel: Real-time qPCR at 48 and 60 h after transfection; (**d**) Silencing of Pokemon in BEL7404 cells. Upper panel: Western blot; lower panel: Real-time qPCR. * *p* < 0.05 compared to the negative control. N.C. means negative control.

We performed gene-specific manipulation of Pokemon expression, focusing on the changes of p38β with ubiquitously existed p38α as a control. We found that ectopic expression of Pokemon increased p38β expression at both mRNA and protein levels in HepG2 cells, but p38α expression was not increased significantly (Figure 4a,b). In sharp contrast, p38β protein was notably reduced by si-RNA-mediated Pokemon silencing (Figure 4a). To further confirm this finding, we also up-regulated Pokemon expression in HL7702 cells and silenced Pokemon in BEL7404 cells, and we got the consistent results (Figure 4c,d). As the close association between ATF2 and p38β [27], the changes of downstream phosphorylated ATF2 also suggested that the ectopic expression or silence of Pokemon efficiently affected p38β activity, by the regulation of Pokemon on p38β expression. Hence, we confirmed that Pokemon only promoted p38β expression in hepatic cells.

### 2.4. Pokemon Promotes p38β Expression by Up-Regulating Its Transcription

**Figure 5:**
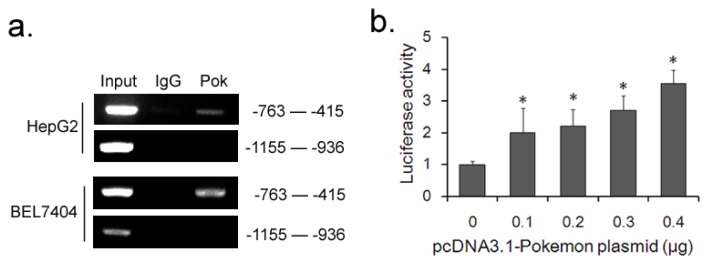
Pokemon stimulates p38β promoter activity. (**a**) ChIP assays in HepG2 and BEL7404 cells. Protein-DNA complexes are immunoprecipitated either with anti-Pokemon antibody or anti-IgG as negative control, followed by PCR with primers specific to p38β promoter sequence and agarose-gel electrophoresis for visualization. Total lysates were used as the input samples and positive control; (**b**) Dual luciferase reporter assay. Luciferase activities were normalized to Renilla activity. Y axis stands for the relative fold changes of activity as the pcDNA3.1(−)-Pokemon plasmid increases. * *p* < 0.05 compared to the negative control.

The effect on p38β mRNA level suggested that Pokemon may regulate p38β expression at the transcriptional level. We next examined whether Pokemon binds to the endogenous p38β promoter *in vivo* using a ChIP assay. Total lysates from HepG2 and BEL7404 cells were used as an input and positive control, while immunoprecipitates with serum IgG served as a negative control. As shown in Figure 5a, Pokemon protein bound to the p38β promoter approximately between −763 bp and −415 bp upstream of the transcriptional start site, but there was no positive signal at more distant upstream region of between −1155 bp and −936 bp. To further investigate whether Pokemon transcriptionally activates p38β promoter, we constructed a luciferase reporter expression plasmid containing wild type p38β promoter sequence, and co-transfected with Pokemon expression plasmid into 293T cells to precede a Dual luciferase report assay. The results indicated that Pokemon indeed enhanced the promoter activity of p38β dose-dependently (Figure 5b).

### 2.5. Discussion

As it is reported in our former ChIP-on-Chip assay, a total of 556 genes were identified as targets of Pokemon in HepG2 cells, being transcriptionally regulated [33]. Among the target genes, we find Pokemon plays a pivotal regulatory role in MAPK signaling pathways. The MAPK11 gene, which encodes p38β, is considered as a potential direct binding target of Pokemon. In this study, we aimed to further clarify the relationship between Pokemon and p38β, and tried to enhance the understanding of the mechanisms by which Pokemon affects cellular biologic behaviors in hepatic cells.

It was reported that Pokemon promotes the growth of ovarian cancer cells [31]. In this study, we confirmed this role of Pokemon in hepatic cells, particularly in the clonogenic growth, a more representative assay of cancer cell tumorigenicity. In the MTT cell growth assays, HepG2 cells with ectopic Pokemon expression demonstrated significant superiority in proliferation at 96 and 120 h, which suggested that overexpressed Pokemon acted as an oncogene and rendered HepG2 cells a growth advantage at high cell density and low nutrition status. However, in HepG2 and BEL7404 cells, silencing of Pokemon did not dramatically affect cell growth. Pokemon may not be a critical oncogenic signal, and possibly other pathways play a dominant role in such state. It is noteworthy to notice that the p38 inhibitor, SB202190, greatly attenuated the cell proliferation stimulated by Pokemon, suggesting its correlation with the p38 signaling pathway.

The p38 signaling pathway is implicated in cellular migration [24]. This study demonstrated that Pokemon acted as an upstream factor of this pathway to promote cell invasion and migration, suggesting Pokemon as a potential indicator of metastatic capability of malignant hepatic cells. In addition, a combination of p38 inhibitor SB202190 with Pokemon silencing additively reduced the invasive capability of HepG2 cells, and a similar phenomenon has been observed in colony formation assays (Figure S3b,d). It has been reported that Pokemon promotes cell metastasis by activating MMP-1 gene in ovarian cancer [31]. Our results revealed a novel regulatory mechanism that Pokemon may regulate the metastasis of hepatic cells through a p38β-dependent pathway.

Many reports suggest that Pokemon plays a transcription factor role in protein expression. We found that Pokemon had a promoting effect on p38β expression and its downstream, phosphorylated ATF2, but no significant effect on p38α (Figure 4). p38 inhibitor SB202190, which specifically inhibits the activity of p38β and p38α, efficiently attenuates the levels of p38β and phosphorylated ATF2 triggered by Pokemon, proving the role of p38α or p38β in this signaling. The affinity of p38β to ATF2 is twenty times larger than that of p38α [27]. Because of the indistinctive change of ATF2 mRNA after Pokemon overexpression, we have reason to believe that the increased level of phosphorylated ATF2 is due to the up-regulation of p38β. Nevertheless, we found only p38β, and not p38α expression, is regulated by Pokemon. According to these results, we confirmed p38β signaling pathway is one of the patterns by which Pokemon modulates. It is noteworthy that a live cell responds to stress in a complicated network, particularly in phosphorylation signaling transition. Therefore, in our studies, the Pokemon protein level was not proportional to the phosphorylation of the effector proteins, and these results of our study provide a potential pattern of Pokemon affecting MAPK signaling pathways.

In our study, we found that p38β, encoded by the MAPK11 gene, is one target of Pokemon in the MAPK signaling pathways, which is consistent with the ChIP-on-Chip results [33]. In summary, our study proposed that in hepatic cells, Pokemon promotes the expression of p38β by directly binding to its promoter region upstream of transcription start site and thus modulates the p38 signaling; whether this is one of the mechanisms through which Pokemon acts as an oncogene and promotes cell growth, migration and invasion will require further research.

## 3. Experimental Section

### 3.1. Cell Culture

Hepatoblastoma-derived cell line HepG2, hepatocellular carcinoma cell line BEL7404, QGY7703, human immortalization hepatic cell line HL7702, Human embryo kidney cell 293T were purchased from the Chinese Academy of Sciences cell bank and cultured in RPMI-1640 or DMEM (High Glucose) medium, supplemented with 10% fetal bovine serum (FBS, Hyclone, Wilmington, DE, USA). Cells were maintained at 37 °C with 5% CO_2_ atmosphere.

### 3.2. Real-Time Quantitative PCR

Pokemon expression vector pcDNA3.1(−)-Pokemon were produced in previous studies [9]. si-RNA was purchased from GenePharma, sequences are: si-Pokemon, 5′-GCU GGA CCU UGU AGA UCA ATT-3′ (forward) and 5′-UUG AUC UAC AAG GUC CAG CTT-3′ (reverse); negative control si-RNA (N.C.), 5′-UUC UCC GAA CGU GUC ACG UTT-3′ (forward) and 5′-ACG UGA CAC GUU CGG AGA ATT-3′ (reverse). Transfection assay was preceded following lipofectamine 2000 protocol (Invitrogen, Grand Island, NY, USA). Total RNA was extracted after transfection for 48 or 60 h using Trizol Reagent (Invitrogen). RNA was reversely transcribed to cDNA using PrimeScript RT Master Mix (TaKaRa, Dalian, Liaoning, China).

DNA was used for quantitative PCR analysis performed. cDNA, primers, and Master Mix (SYBR Premix Ex TaqTM, TaKaRa), were mixed as the instruction of manufacturer. Reactions were performed by a 7500 Fast Real-time PCR System (Applied Biosystems, Bedford, OH, USA). Primers were as follows: p38α: 5′-TGG AAG CCT GGA CTC TAA-3′ (forward) and 5′-ATC CTA TAC GGC ATA ACT G-3′ (reverse); p38β: 5′-AGA AGG TGG CGG TGA AGA-3′ (forward) and 5′-CGT CCA GAA GCC CGA TGA-3′ (reverse); GAPDH: 5′-GGT GGT CTC CTC TGA CTT CAA CA-3′ (forward) and 5′-GTT GCT GTA GCC AAA TTC GTT GT-3′ (reverse); and Pokemon: 5′-GAA GCC CTA CGA GTG CAA CAT C-3′ (forward) and 5′-GTG GTT CTT CAG GTC GTA GTT GTG-3′ (reverse).

### 3.3. Western Blot

Proteins were extracted from treated cells and separated in 10%–12% SDS-polyacrylamide gel and then transferred to a PVDF membrane (Amersham Bioscience, Piscataway, NJ, USA). The membranes were blocked in 5% bovine serum albumin (BSA, Sangon Biotech. Shanghai, China) and then incubated overnight at 4 °C in first antibody. The membranes were washed and incubated with horseradish peroxidase-conjugated second antibody. Protein bands were visualized by an imaging system (Bio-Rad, Munich, Germany) after developed with ECL reagents (Thermo Scientific, Wilmington, DE, USA). Antibodies used are as follows: Pokemon antibody (Sigma, St. Louis, MO, USA), p38α MAPK (7D6) Rabbit mAb (CST, Danvers, MA, USA), p38β MAPK (c28c2) Rabbit mAb (CST), Phospho-p38 MAPK Pathway Sampler Kit (CST) and Actin antibody (Beyotime, Shanghai, China).

### 3.4. Dual Luciferase Reporter Assay

We constructed wide-type promoter plasmid pGL4.10-p38β, empty pGL4.10 was used as a negative control. Primers for promoters are 5′-CCC GGT ACC GCT GGC TGT TTT AAT TTG G -3′ (forward) and 5′-TAA CTC GAG AGC TCC TGC CGG TAG AAG-3′ (reverse), with KpnI and XhoI restriction enzyme cut sites.

This assay was preceded in 293T cells because of their high transfection efficiency. After transfection for 36 h, cells were dissolved with passive lysis buffer (1×). Luciferase activity was measured using a Dual luciferase reporter assay kit (Promega, Fitchburg, WI, USA).

### 3.5. Chromatin Immunoprepcipitation (ChIP)

ChIP assay was preceded according to ChIP-IT^®^ express enzymatic magnetic chromatin immunoprecipitation kit & enzymatic shearing kit protocols. DNA fragments were used as templates to preced PCR. Primers are as follows: Between −763 bp and −415 bp: 5′-TGG AGG GGG TCG CCC AGC CGC GAA G-3′ (forward) and 5′-TGC CCA GAA CCT TTC CTC CT-3′ (reverse); between −1155 bp and −936 bp: 5′-GGA CTG AGA CCC GTT CCT TCG-3′ (forward) and 5′-TGG TAG AGC CGT GGT GGG AG-3′ (reverse). Antibodies used are as follows: Anti-Pokemon antibody (Abcam, Cambridge, UK) and Goat Control IgG antibody (Abcam).

### 3.6. MTT Assay

Cells were seeded at a density of 3 × 10^3^/100 μL medium in 96-well plate and treated with the SB202190 (Merck Millipore, Darmstadt, Germany) at different time points, ranging from 24 to 120 h. Cells were incubated with MTT (5 mg/mL) for 4 h and formazan precipitate was dissolved in 100 μL DMSO and the absorbance at 595 nm was measured by Multimode Detector DTX880 (Beckman Coulter, Atlanta, GA, USA).

### 3.7. Colony Formation Assay

HepG2 cells at the exponential phase were plated into 24-well plate (200–300 cells/well) and allowed to adhere for 12 h before treatment. Then the culture medium was substituted with fresh medium containing SB202190 and incubated for 14 days. Cells were then counted and photographed after rinsed with PBS and stained with 1.0% crystal violet.

### 3.8. Invasion and Migration Assays

The invasion ability of cells was measured by the Boyden chamber invasion assay. Matrigel (Sigma) was diluted at the ratio of 1:1 with pre-cooled culture medium and added on the upper chamber of the 8 μm pore polycarbonate filter (Merck Millipore). The medium containing 10% FBS was added in the lower chamber, and the treated cells were re-suspended in serum-free medium and plated on the upper chamber. After 24 h incubation at 37 °C, the cells in the upper surface were carefully removed with a cotton swab and cells invaded across the matrigel were stained with crystal violet, counted and photographed.

The migration assay was preceded similarly as the invasion assay described above, except the upper chamber of polycarbonate filters were not coated with matrigel.

### 3.9. Statistical Analysis

All Data were presented as mean ± S.E.M. from experiments done in triplicate. Statistical analyses were performed using Student’s *t*-test. The difference was considered statistically significant when the *p* value was <0.05.

## 4. Conclusions

Pokemon is considered a pivotal transcription factor, involved in multiple biological processes [12,13] and localized upstream of several oncogenes [17]. In this study, we confirmed the promoting effect of Pokemon on cell growth, migration and invasion in hepatic cells. Through the attenuation of the promotion by SB202190, we uncovered a new mechanism that Pokemon is involved in. Pokemon transcriptionally up-regulates the expression of p38β and consequently stimulates p38 signaling.

## Supplementary Information
