# Supplementary material for: p38β, A Novel Regulatory Target of Pokemon in Hepatic Cells
Source: Int J Mol Sci. 2013 Jun 27;14(7):13511–24. doi: 10.3390/ijms140713511 (PMC3742200; doi:10.3390/ijms140713511)
Supplement: Supplementary file 1 [file ijms-14-13511-s001.pdf]

# Supplementary Information

**Figure S1.** Cell growth rate by the MTT assay. (**a**) HL7702 cells were transfected with pcDNA3.1(−)-Pokemon (marked as Pok) or pcDNA3.1(−) (marked as 3.1); (**b**) Pokemon was silenced by si-RNA (marked as si-Pok) or scramble RNA (marked as N.C.) in BEL7404 cells. * $p < 0.05$ compared to the negative control group.

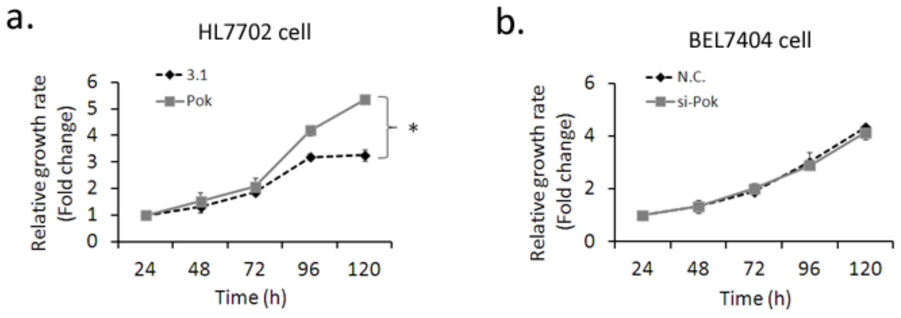


**Figure S2.** Silencing Pokemongene on BEL7404 cells metastasis. (**a**) *In vitro* migration assays; (**b**) *In vitro* invasion assays. Bar chart below the photo stands for the relative fold of the migrated or invaded cell number compared to the negative control group. * $p < 0.05$ compared to the negative control group.

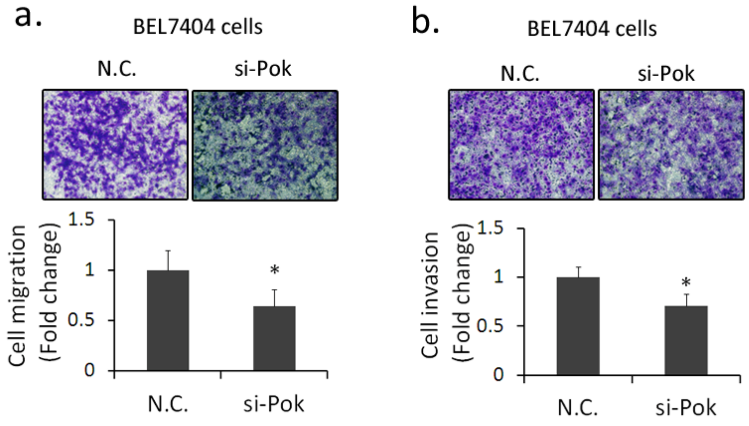

**Figure S3.** (**a**) HL7702cell growth rate: Cells were treated with 25 μM SB202190 at 24 h after transfecting with pcDNA3.1(−)-Pokemon or pcDNA3.1(−); (**b**–**d**) Effect of silencing Pokemongene and p38 inhibitor SB202190 on colony formation, migration and invasion in HepG2 cells: (**b**) The colony formation assay, the rate below the photostands for the proportion of final clone number accounted for in plated cell number; (**c**) *In vitro* migration assays; (**d**) *In vitro* invasion assays. Bar chart below the photo stands for the relative fold of the migrated or invaded cell number compared to the negative control group. * $p < 0.05$ compared to the negative control group.

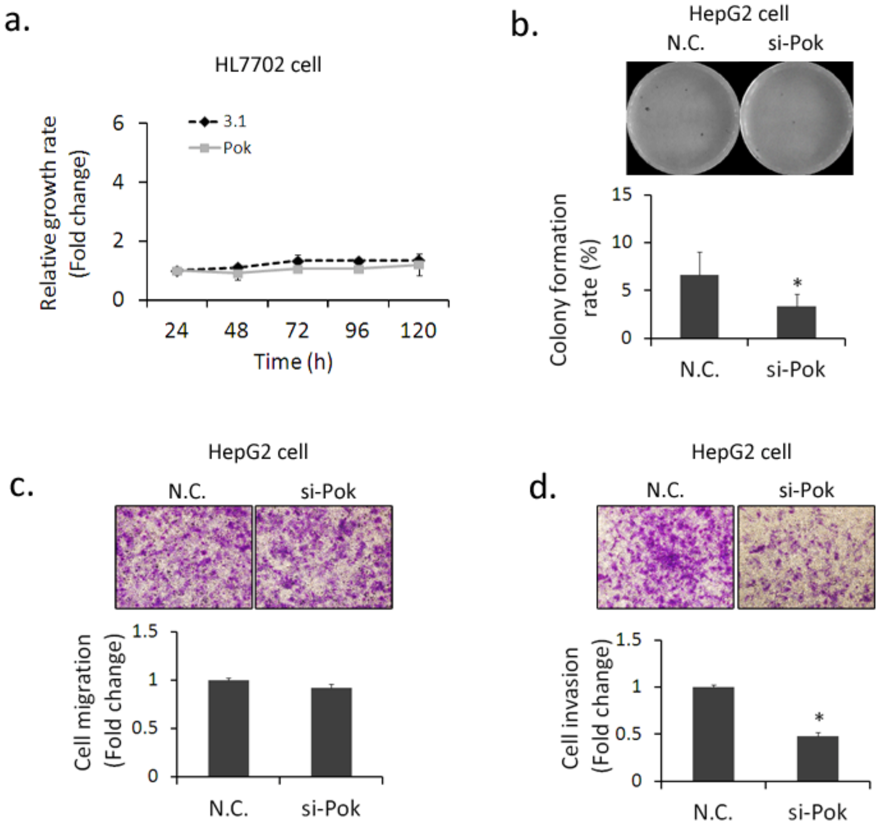


© 2013 by the authors; licensee MDPI, Basel, Switzerland. This article is an open access article distributed under the terms and conditions of the Creative Commons Attribution license (http://creativecommons.org/licenses/by/3.0/).
